# Establishing the Optimal Ceftriaxone MIC for Predicting ESBL Production in Bloodstream Infections

**DOI:** 10.1093/ofid/ofaf490

**Published:** 2025-08-14

**Authors:** Dariusz A Hareza, Yehudit Bergman, Emily Jacobs, Amy J Mathers, Alan R Hauser, Sara E Cosgrove, Patricia J Simner, Pranita D Tamma

**Affiliations:** Department of Medicine, Johns Hopkins University School of Medicine, Baltimore, Maryland, USA; Department of Pediatrics, Johns Hopkins University School of Medicine, Baltimore, Maryland, USA; Department of Pathology, Johns Hopkins University School of Medicine, Baltimore, Maryland, USA; Department of Pediatrics, Johns Hopkins University School of Medicine, Baltimore, Maryland, USA; Department of Pathology, Johns Hopkins University School of Medicine, Baltimore, Maryland, USA; Department of Medicine, University of Virginia Health System, Charlottesville, Virginia, USA; Department of Microbiology-Immunology, Northwestern University, Chicago, Illinois, USA; Department of Medicine, Johns Hopkins University School of Medicine, Baltimore, Maryland, USA; Department of Medicine, Johns Hopkins University School of Medicine, Baltimore, Maryland, USA; Department of Pathology, Johns Hopkins University School of Medicine, Baltimore, Maryland, USA; Department of Pediatrics, Johns Hopkins University School of Medicine, Baltimore, Maryland, USA

To the  Editor—Extended-spectrum β-lactamase-producing Enterobacterales (ESBL-E) hydrolyze most β-lactam antibiotics, with the general exception of carbapenems [1]. CTX-M enzymes constitute the vast majority of ESBLs and are predominantly produced by *Escherichia coli* and *Klebsiella pneumoniae* [1, 2]. While an increasing number of United States hospitals are adopting molecular platforms to detect *bla*_CTX-M_ genes in blood cultures, routine testing remains inconsistent, particularly for specimens from other sites (eg, urine).

Consequently, and in line with the IDSA Antimicrobial Resistance Treatment Guidance [3], clinicians often infer ESBL production (in the absence of ESBL confirmatory testing) based on non-susceptible results to ceftriaxone, defined as a ceftriaxone minimum inhibitory concentrations (MIC) of ≥2 μg/mL (ie, intermediate or resistant). While this threshold offers high sensitivity (ie, most ESBL-E isolates have ceftriaxone MICs ≥2 μg/mL), it lacks specificity, as a portion of *E. coli* and *K. pneumoniae* isolates with ceftriaxone MICs at or above this level do not produce ESBLs. Notably, there is no Clinical and Laboratory Standards Institute (CLSI) recommendation to use the ceftriaxone MIC as a surrogate for ESBL production [4].

Employing an overly sensitive ceftriaxone MIC threshold to infer ESBL production leads to overuse of carbapenems, the preferred treatment for invasive ESBL-E infections [5]. This approach may exacerbate the expanding crisis of carbapenem resistance [6]. Nevertheless, we believe sensitivity should be prioritized over specificity as failing to identify an ESBL-producing isolate could result in suboptimal patient outcomes [3].

We sought to determine the optimal ceftriaxone MIC threshold that could serve as a surrogate for ESBL production. We analyzed a cohort of *E. coli* and *K. pneumoniae* bloodstream isolates that were non-susceptible to ceftriaxone (MIC ≥2 μg/mL) but susceptible to carbapenems, based on routine antimicrobial susceptibility testing (AST) using the BD Phoenix™ automated identification and susceptibility testing system. Tested isolates were collected from unique, consecutive patients at the Johns Hopkins Health System between 2018 and 2022. AST was confirmed for all isolates using Sensititre broth microdilution (BMD) GN2F panels (Thermo Fisher Scientific), following CLSI criteria [4]. ESBL genes were identified using Illumina whole genome sequencing, using methods previously described [2]. ESBL genes were classified according to the Bacterial Antimicrobial Resistance Reference Gene Database [7].

A receiver operating characteristic (ROC) curve was constructed to determine the optimal ceftriaxone MIC threshold for ESBL production. ROC analysis was conducted with the “roctab” function in Stata 17. The area under the curve (AUC) was calculated to assess the ability of ceftriaxone MICs to distinguish ESBL positive and negative isolates, with values ≥0.80 indicative of good discriminatory performance [8].

Three hundred eighty-seven *E. coli* and *K. pneumoniae* isolates met inclusion criteria; 362 (94%) contained at least one ESBL gene. Ceftriaxone MIC distributions and ESBL genes identified are displayed in Table 1. More specifically, 265 of the 287 (92%) *E. coli* isolates and 97 of the 100 (97%) *K. pneumoniae* isolates contained an ESBL gene. Of note, 23 (8%) and 2 (2%) of *E. coli* and *K. pneumoniae* isolates, respectively contained a plasmid-mediated *ampC* gene (*bla*_CMY-59_ or *bla*_DHA-1_). The median ceftriaxone MIC for isolates containing an ESBL gene was 128 µg/mL (IQR 128–128 µg/mL). The ROC curve yielded an AUC of 0.87. Ceftriaxone MIC cutoffs of 8 μg/mL, 16 μg/mL, 32 μg/mL, 64 μg/mL, and ≥128 µg/mL yielded sensitivities of 99%, 98%, 96%, 94%, and 85%, respectively, for ESBL detection. These findings align with prior studies indicating that the majority of ESBL-producing isolates exhibit ceftriaxone MICs above 32 μg/mL [9–11]. Our study suggests the commonly used ceftriaxone MIC threshold of ≥2 μg/mL for ESBL detection lacks specificity; a cutoff of 8 µg/mL or higher may be more appropriate.

**Table 1.** Distribution of Ceftriaxone MICs and ESBL Gene Status Amongst 387 Escherichia Coli and Klebsiella Pneumoniae Bloodstream Isolates Non-susceptible to Ceftriaxone Collected From Blood Cultures From Consecutive, Unique Patients^a^

Ceftriaxone MIC (µg/mL)	Total # of Isolates	# of Isolates with ≥1 ESBL Genes^b^	ESBL Genes	Plasmid-mediated *ampC* Genes
2	8	1	*bla*_SHV-7_ (*n* = 1)	*bla*_DHA-1_ (*n* = 1)
4	6	2	*bla*_SHV-7_ (*n* = 1), *bla*_SHV-30_ (*n* = 1)	*bla*_CMY-59_ (*n* = 1)
8	12	3	*bla*_CTX-M-15_ (*n* = 3)	*bla*_DHA-1_ (*n* = 1), *bla*_CMY-59_ (*n* = 1)
16	12	7	*bla*_CTX-M-15_ (*n* = 4), *bla*_CTX-M-27_ (*n* = 1), *bla*_SHV-39_ (*n* = 1), *bla*_SHV-187_ (*n* = 1)	*bla*_CMY-59_ (*n* = 5)
32	10	10	*bla*_CTX-M-3_ (*n* = 1), *bla*_CTX-M-14_ (*n* = 1), *bla*_CTX-M-15_ (*n* = 3), *bla*_CTX-M-27_ (*n* = 1), *bla*_SHV-2_ (*n* = 3), *bla*_SHV-187_ (*n* = 1)	*bla*_CMY-59_ (*n* = 2), *bla*_DHA-1_ (*n* = 1)
64	31	31	*bla*_CTX-M-14_ (*n* = 2), *bla*_CTX-M-15_ (*n* = 18), *bla*_CTX-M-27_ (*n* = 11)	*bla*_CMY-50_ (*n* = 4), *bla*_DHA-1_ (*n* = 1)
≥128	308	308	*bla*_CTX-M-1_ (*n* = 1), *bla*_CTX-M-3_ (*n* = 4), *bla*_CTX-M-14_ (*n* = 14), *bla*_CTX-M-15_ (*n* = 238), *bla*_CTX-M-27_ (*n* = 41), *bla*_CTX-M-55_ (*n* = 8), *bla*_CTX-M-55_ + *bla*_SHV-187_ (2)	*bla*_CMY-59_ (*n* = 5), *bla*_DHA-1_ (3)

^a^All antibiotic MICs derived from broth microdilution and ESBL gene identification derived from whole genome sequencing.

^b^Out of 362 isolates with ≥1 ESBL genes.

Our findings should not be interpreted as a call to revise ceftriaxone breakpoints. Antibiotic breakpoints are based on several factors including wild-type MIC distributions, pharmacokinetic/pharmacodynamic models, and clinical outcomes data [12]. For example, in a person with an invasive *E. coli* infection with a ceftriaxone MIC of 4 μg/mL, our data suggest the likelihood of ESBL production is low. However, this does not imply that treatment with the standard ceftriaxone dosage of 1 g every 24 h would be appropriate. Achieving the necessary ceftriaxone pharmacokinetic/pharmacodynamic target for efficacy (ie, fT > MIC of ≥50%) [13] would be highly unlikely for isolates with ceftriaxone MICs of 4 µg/mL, potentially leading to poor patient outcomes. In such cases, although ceftriaxone is a suboptimal choice, meropenem may be an unnecessary choice. Agents like cefepime or piperacillin-tazobactam (if susceptible) could be viable alternatives [3]. Notably, all *E. coli* and *K. pneumoniae* isolates with ceftriaxone MICs ≤8 µg/mL in our cohort were susceptible or susceptible dose-dependent to both piperacillin-tazobactam and cefepime, based on BMD results.

Given the poor discriminatory ability of ceftriaxone MICs ≥2 μg/mL in differentiating ESBL producers from non-producers, we believe that reconsidering this surrogate threshold commonly used by clinicians may improve carbapenem use. The isolates included in this study came from a limited geographic region (ie, the mid-Atlantic United States). Additionally, only blood culture isolates were included. It is unclear if ceftriaxone MIC distributions and the presence of ESBL genes differ by site of infection. This work needs to be repeated in a larger, more diverse cohort with isolates from other sites of infection.
